# Inhibition of Bacterial RNase P RNA by Phenothiazine Derivatives

**DOI:** 10.3390/biom6030038

**Published:** 2016-09-08

**Authors:** Shiying Wu, Guanzhong Mao, Leif A. Kirsebom

**Affiliations:** Department of Cell and Molecular Biology, Box 596, Biomedical Centre, Uppsala SE-751 24, Sweden; littleyaya830@icloud.com (S.W.); guanzhong.mao@icm.uu.se (G.M.)

**Keywords:** RNA processing, catalytic RNA, RNase P, phenothiazines

## Abstract

There is a need to identify novel scaffolds and targets to develop new antibiotics. Methylene blue is a phenothiazine derivative, and it has been shown to possess anti-malarial and anti-trypanosomal activities. Here, we show that different phenothiazine derivatives and pyronine G inhibited the activities of three structurally different bacterial RNase P RNAs (RPRs), including that from *Mycobacterium tuberculosis*, with *K_i_* values in the lower μM range. Interestingly, three antipsychotic phenothiazines (chlorpromazine, thioridazine, and trifluoperazine), which are known to have antibacterial activities, also inhibited the activity of bacterial RPRs, albeit with higher *K_i_* values than methylene blue. Phenothiazines also affected lead(II)-induced cleavage of bacterial RPR and inhibited yeast tRNA^Phe^, indicating binding of these drugs to functionally important regions. Collectively, our findings provide the first experimental data showing that long, noncoding RNAs could be targeted by different phenothiazine derivatives.

## 1. Introduction

Ribonuclease P (RNase P) is an essential and ubiquitous endoribonuclease responsible for generating tRNAs with matured 5′ ends. In Bacteria, RNase P is composed of an approximately 400 nucleotides-long RNA (RPR) and one small basic protein, while in Archaea and Eukarya several proteins associate with the sole RNA. Irrespective of origin, the catalytic moiety of RNase P is the RPR, as evident from its ability to mediate cleavage of pre-tRNA as well as various other natural RNAs including mRNA and artificial substrates without the protein cofactor(s) [1,2,3,4]. Comparison of the bacterial and human RPR reveals structural differences in regions of functional importance. For example, the human RPR lacks the region in bacterial RPR that interacts with the 3′ end of pre-tRNAs; differences also exist in the region that constitutes the binding site for the pre-tRNA T-stem/loop in bacterial RPR [5]. In addition, 10 proteins bind to human RPR compared to 1 in bacteria [6]. Together, these findings suggest that bacterial RNase P is a promising target for new anti-infective agents [7,8,9,10,11,12,13].

The activity of bacterial RNase P is inhibited by various aminoglycosides, aminoglycoside–arginine conjugates, and puromycin [10,14,15,16,17]. Biochemical and structural data suggest that aminoglycosides can compete with binding of functionally important Me(II)-ion(s) to RNA [15,17,18]. In addition, acitretin and aminoglycosides inhibit the activity of the *Dictyostelium discoideum* RNase P holoenzyme [19,20]. Thus, we were motivated to search for other small molecules that interfere with bacterial RNase P function. In this study, we focused on bacterial RPR activity without protein since we were interested in identifying inhibitors of RNA function.

The phenothiazine methylene blue was first synthesized and described in the late 1800s. Given its properties, it was used as both a stain and a drug for the treatment of bacterial infections and malaria. Its biochemical properties have also been instrumental in developing protocols for microscopic detection and identification of microorganisms such as *Mycobacterium tuberculosis*, the causative agent of tuberculosis [21]. Moreover, staining procedures involving methylene blue have been used to stain RNAs in tissues and for visualization of RNA following separation by gel electrophoresis and membrane blotting [22,23].

Here, we provide data showing that various phenothiazine derivatives such as toluidine blue O and chlorpromazine, an antipsychotic drug with antibacterial activity, inhibit the activity of three structurally different bacterial RPRs, including the *M. tuberculosis* variant, as well as lead(II)-induced cleavage of yeast tRNA^Phe^.

## 2. Results

**Figure 1:**
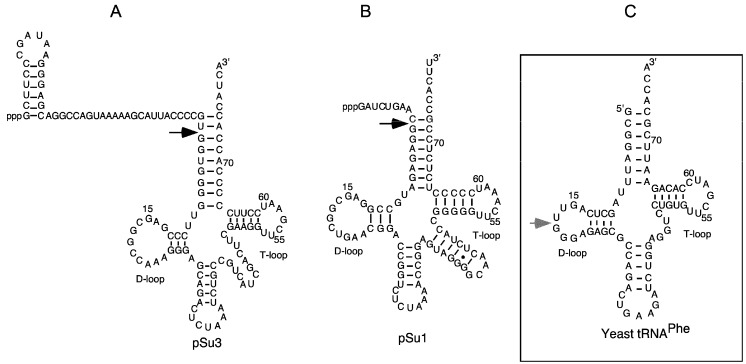
The secondary structures of precursor tRNA^Tyr^Su3, precursor tRNA^Ser^Su1, and yeast tRNA^Phe^. (**A**,**B**) Precursor tRNA^Tyr^Su3 (pSu3) and tRNA^Ser^Su1 (pSu1). Black arrows indicate the canonical RNase P cleavage sites; (**C**) The grey arrow marks the major Pb^2+^-induced cleavage site in the D-loop of yeast tRNA^Phe^.

**Figure 2:**
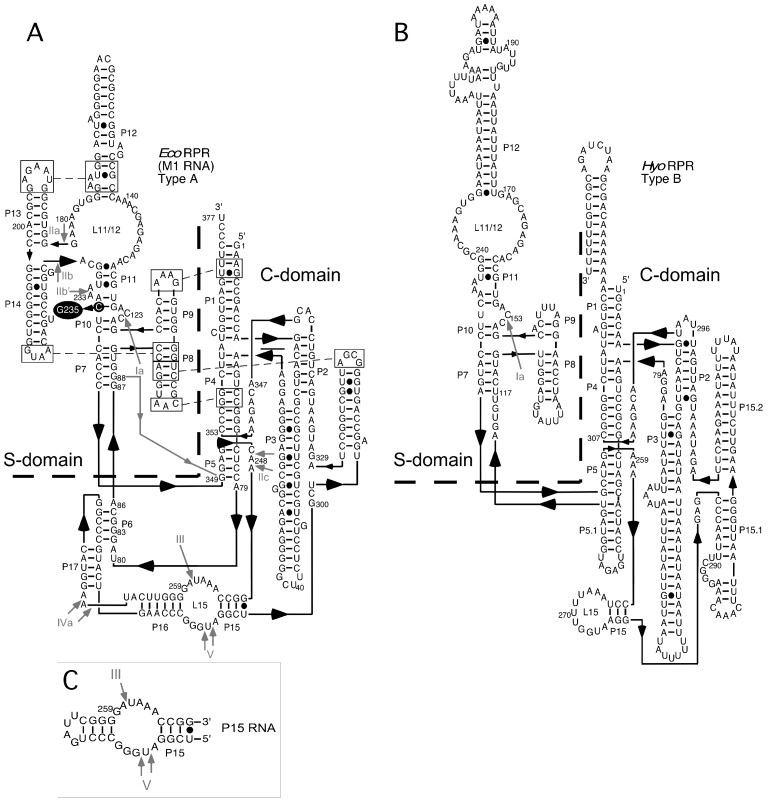
The secondary structures of *Escherichia coli* (*Eco*) RNase P RNA (RPR) (type A), *Mycoplasma hyopneumoniae* (*Hyo*) RPR (type B), and P15 RNA according to Massire et al. [29]. (**A**) *Eco* RPR (type A), the dotted line demarcates the S- and C-domains. Roman numerals and grey arrows mark the Pb^2+^-induced cleavage sites I to V. Residue C_235_ highlighted in black was mutated to G to generate *Eco* RPR_G235_. The *Eco* CP RPR_wt_ lacks the S-domain and constitutes only the C-domain, with A_79_ to G_88_ forming a loop, marked with a grey line/arrow; for details see Kikovska et al. [30] and Wu et al. [31]; (**B**) *M. hyopneumoniae* RPR (type B) [26]. The Ia Pb^2+^-induced cleavage site is marked with a grey arrow. As in A, the dotted line demarcates the two major RPR domains; (**C**) P15 RNA. The grey arrow marks the Pb^2+^-induced cleavage at sites III to V.

**Figure 3:**
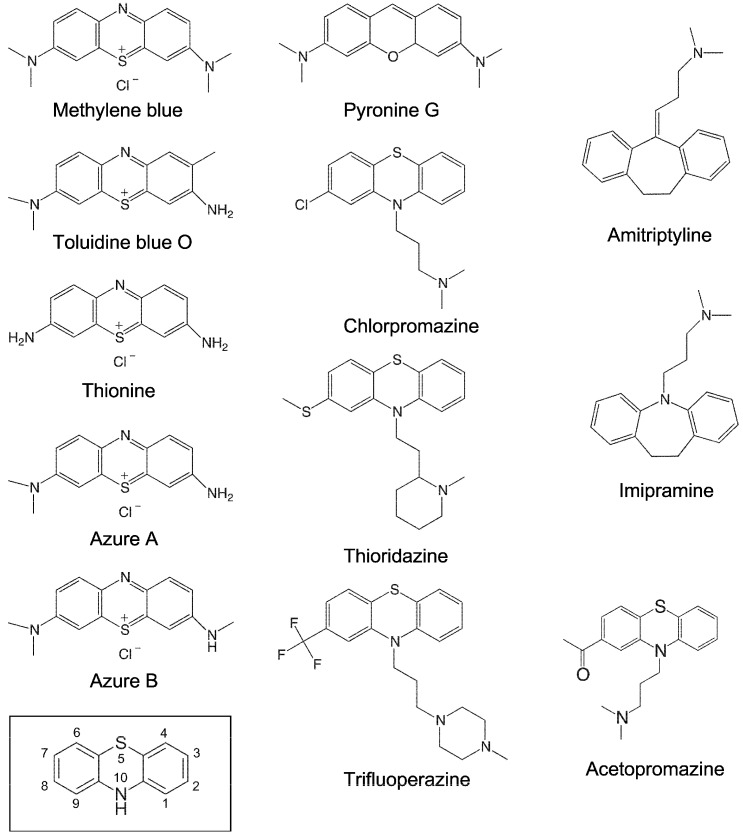
Chemical structures of the small molecules tested in this study. The structure marked with a box represents the phenothiazine scaffold structure, with the ring atoms numbered.

To investigate whether phenothiazine derivatives inhibit the activity of bacterial RPR we followed the protocol described by Mikkelsen et al. ([15], see Materials and Methods) and monitored cleavage of *Escherichia coli* (*Eco*) pre-tRNA^Tyr^Su3 (pSu3) and pre-tRNA^Ser^Su1 (pSu1; Figure 1). Bacterial RPRs have been classified into type A (ancestral) and type B (Bacillus type) based on their secondary structures ([24]; Figure 2). Hence, we decided to use *Eco* RPR_wt_ (M1 RNA; type A) and also *Eco* RPR_G235_, which carries a change that alters the structure in the vicinity of the binding site for the pre-tRNA T-stem/loop region ([25]; see below), and *Mycoplasma hyopneumoniae* (*Hyo*) RPR (type B; Figure 2; [26]). In addition, we included the type A *M. tuberculosis* (*Myc*) RPR because of its medical importance and the alarming increase in the appearance of multidrug-resistant strains (Figure 2; [27,28]). The structures of the different phenothiazine derivatives and similar compounds that we tested are depicted in Figure 3.

### 2.1. Inhibition of RNase P RNA Cleavage by Phenothiazine Derivatives

**Figure 4:**
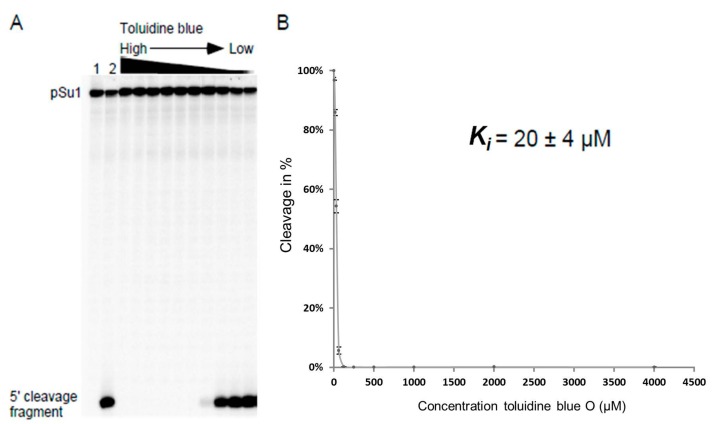
Toluidine blue inhibition of *Eco* RPR_wt_-mediated cleavage of pSu1. (**A**) A representative experiment showing toluidine blue inhibition of *Eco* RPR_wt_ activity. The experiment was performed as outlined in the Materials and Methods and the reaction time was 5 min. Lanes 1 and 2 correspond to controls; lane 1 corresponds to pSu1 substrate without *Eco* RPR_wt_ and toluidine blue and lane 2 corresponds to pSu1 with *Eco* RPR_wt_ but without toluidine blue; (**B**) Efficiency of cleavage is expressed in percentage as a function of increasing concentration of toluidine blue.

Increasing concentrations of the selected phenothiazine derivatives inhibited the activity of all three bacterial RPRs. A representative inhibition curve is shown in Figure 4 and the *K_i_* values were determined to be in the low μM range irrespective of the RPR we tested (Table 1). Comparing the *K_i_* values for *Eco* RPR_wt_, *Myc* RPR and *Hyo* RPR revealed little variation, at most twofold. Replacing pSu3 with pSu1 in our assays resulted in a twofold increase in *K_i_* for inhibition of *Eco* RPR_wt_ by toluidine blue O (cf. Table 1 and Table 2). These data demonstrated that the addition of phenothiazine compounds inhibits the activity of structurally different bacterial RPRs.

The intercalator RNA dye pyronine G, which has a similar structure compared to phenothiazine (Figure 3), also inhibited the activity of all three RPRs in the low μM range, with a threefold variation in *K_i_* (Table 1). Pyronine G has an oxygen and a carbon at positions 5 and 10, respectively, while phenothiazine has sulfur and nitrogen at those positions (Figure 3). Thus, having sulfur and nitrogen at positions 5 and 10 is not essential for inhibiting the activity of bacterial RPRs.

The inhibition of *Eco* RPR activity by aminoglycosides is pH-dependent [15]. We therefore inquired whether pH also influences the inhibition by phenothiazine. For toluidine blue O, we did not detect any change in *K_i_* when the reaction was performed at pH 6, pH 7.9 (our standard pH), or pH 9 (Table 3). We also inquired whether higher salt concentrations influenced inhibition by toluidine blue O and thionine acid. The *K_i_* values increased two- to almost fourfold in the presence of 100 mM Mg^2+^ and 100 mM NH_4_^+^, i.e., 10 to 100 times higher concentrations, respectively, compared to 10 mM Mg^2+^ and 1 mM NH_4_^+^ (see Materials and Methods). Based on this data alone, however, we cannot determine whether the increase in *K_i_* is due to the higher concentration of NH_4_^+^ and/or the higher concentration of Mg^2+^.

Next, we asked whether the order of addition of *Eco* RPR vs. substrate (S; pSu3) and inhibitor (I; toluidine blue O or thionine acid), had any effect on *K_i_*. The data showed that pre-incubating pSu3 together with the I resulted in at least a fourfold lower *K_i_* than when the RPR and I were pre-incubated together or when RPR, S, and I were pre-incubated separately and then mixed (Table 3). These results indicated that toluidine blue O or thionine acid might bind to pre-tRNA.

### 2.2. Lead(II)-Induced Cleavage of Yeast tRNA^Phe^, P15 RNA, and *Eco* RPR

To test whether phenothiazine indeed binds to tRNA, we studied lead(II)-cleavage of yeast tRNA^Phe^ based on the precedent that aminoglycosides interfere with Pb^2+^ binding to RNA ([15,18]; see Materials and Methods). Our data indicate that all phenothiazine derivatives inhibit lead(II)-cleavage of yeast tRNA^Phe^ (Table 1). The *K_i_* values for inhibition of Pb^2+^-cleavage were higher compared to values determined for inhibiting the bacterial RPRs. In addition, we tested pyronine G and it also inhibited lead(II)-cleavage of yeast tRNA^Phe^. However, *K_i_* was significantly higher than for the phenothiazine derivatives. Together, these data suggested that phenothiazine and pyronine G bind to tRNA, albeit with different affinities.

The functionally important P15 domain in *Eco* RPR_wt_ (type A; Figure 2; [1]) is cleaved at specific sites in the presence of Pb^2+^ in the context of both full-length *Eco* RPR_wt_ and as a separate module [32]. Hence, we asked whether lead(II)-induced cleavage of P15 RNA, a 31-nt-long RNA [33] representing the P15-domain, is also inhibited by phenothiazine and pyronine G. *K_i_* values ranging from 14 to 60 μM indicate that these inhibitors also bind to the P15 RNA. Collectively, these data suggested that phenothiazines bind to RNA and interfere with binding and/or positioning of Pb^2+^.

### 2.3. Inhibition of Bacterial RPR Activities by Antipsychotic Phenothiazine Variants

The antipsychotic drugs thioridazine, trifluoperazine and chlorpromazine are phenothiazine variants with different functional (R) groups, R_1_ and R_2_, at positions 2 and 10 (Figure 3). As was observed for the other phenothiazine derivatives, addition of any of these molecules inhibited the activity of all three RPRs. However, the *K_i_* values, which ranged from 67 to 437 μM, were higher than the values for the other phenothiazine derivatives (Table 1; see also Table 2 where pSu1 was used as substrate). This increased *K_i_* might be due to the presence of bulky R_2_ groups. Moreover, thioridazine, trifluoperazine, and chlorpromazine also inhibited lead(II)-induced cleavage of P15 RNA, with *K_i_* values similar to the values for inhibition of RPR activity. We also tested if the “three ring” antidepressants imipramine and amitriptyline (Figure 3) showed any inhibitory effect on bacterial RPR activity, but we did not detect any inhibition under our conditions (data not shown).

### 2.4. Binding of Phenothiazine Affects the Structure of *Eco* RPR

**Figure 5:**
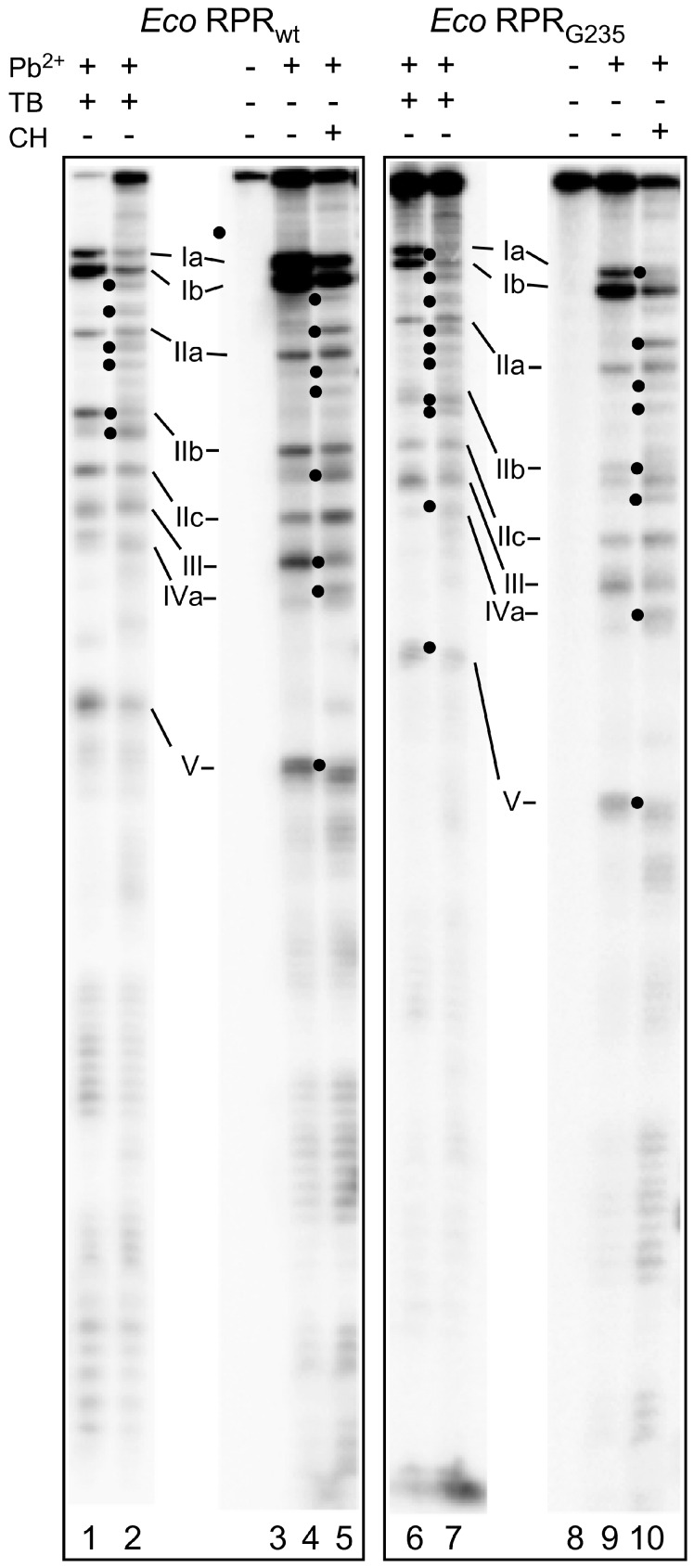
Lead(II)-induced cleavage patterns of 3′-[^32^P]-pCp labeled *Eco* RPR_wt_ and *Eco* RPR_G235_ with and without toluidine blue (TB) or chlorpromazine (CH). The experiments were performed at 37 °C as outlined in the Materials and Methods. The Roman numerals refer to the lead(II)-induced cleavage sites as shown in Figure 2 and the bands marked with • indicate observed changes induced in the presence of toluidine blue. In all the experiments, we used 1 μM RPR. Lane 1: *Eco* RPR_wt_, 0.025 mM toluidine blue and 1 mM Pb^2+^, reaction time 7 min; lane 2: *Eco* RPR_wt_, 0.25 mM toluidine blue and 1 mM Pb^2+^, reaction time 7 min; lane 3: incubation of *Eco* RPR_wt_ without toluidine blue and Pb^2+^, reaction time 7 min; lane 4: *Eco* RPR_wt_ cleaved with Pb^2+^ (2 mM) for 7 min; lane 5: *Eco* RPR_wt_, 4 mM chlorpromazine and 2 mM Pb^2+^, reaction time 7 min; lane 6: *Eco* RPR_G235_, 0.025 mM toluidine blue and 1 mM Pb^2+^, reaction time 7 min; lane 7: *Eco* RPR_G235_, 0.25 mM toluidine blue and 1 mM Pb^2+^, reaction time 7 min; lane 8: incubation of *Eco* RPR_G235_ without inhibitor and Pb^2+^, reaction time 7 min; lane 9: *Eco* RPR_G235_ cleaved with Pb^2+^ (1 mM) for 5 min; lane 10: *Eco* RPR_G235_, 4 mM chlorpromazine and 1 mM Pb^2+^, reaction time of 5 min.

To elucidate where phenothiazine binds RPR_wt_ we used Pb^2+^, which induces cleavage at specific and well-defined positions in the *Eco* RPR_wt_ (Figure 2). As shown in Figure 5, Pb^2+^-induced cleavage of *Eco* RPR_wt_ in the presence of toluidine blue O and chlorpromazine resulted in changes in the vicinities of sites Ib, IIa, and IIb as well as near III, IV, and V in the case of chlorpromazine (Figure 5, marked with •). The change in cleavage at III and V is consistent with phenothiazine inhibition of lead(II)-induced cleavage of P15 RNA (Table 1). Sites IIa and IIb are located in the S domain, which constitutes the binding site for the pre-tRNA T-stem/loop in bacterial RPR [1,34]. Hence, these data suggested that toluidine blue O and chlorpromazine bind to *Eco* RPR, altering its structure and interfering with the binding of Pb^2+^ ions in several *Eco* RPR regions.

### 2.5. Phenothiazine Inhibition of Structurally Altered *Eco* RPR

**Figure 6:**
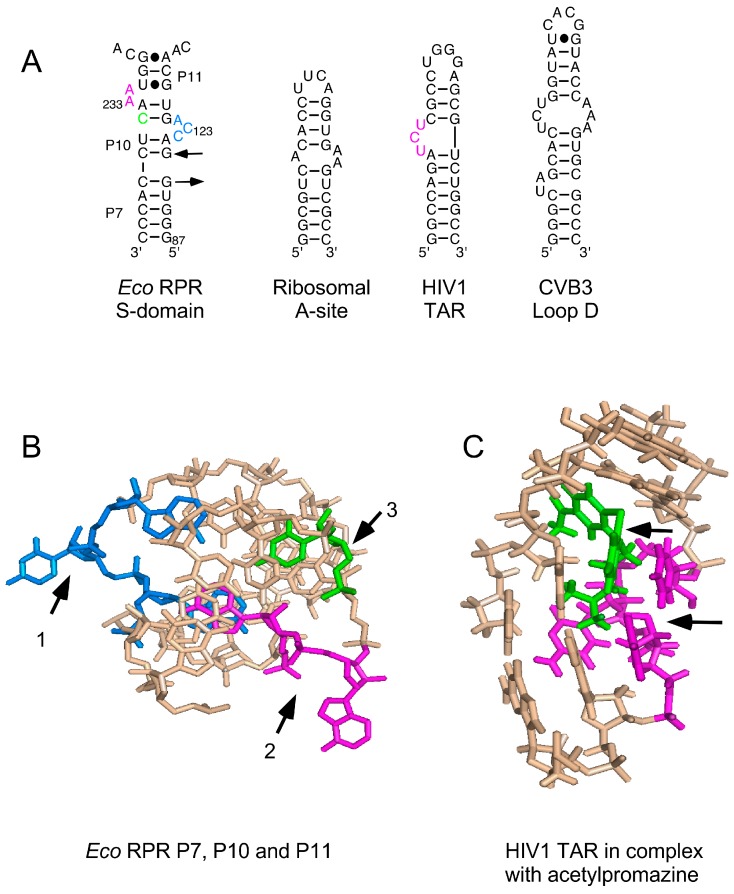
(**A**) Secondary structures of various RNAs shown to bind to phenothiazine; (**B**) Three-dimensional structure of the P7, P10 and P11 region in the S domain that is suggested to constitute one binding site for phenothiazine derivatives. Residues marked with black arrows 1–3 and in blue (C_122_, C_123_ and A_124_), magenta (A_232_ and A_233_) and green (C_235_, changed to G_235_, see Figure 2); for color code see panel A; (**C**) The nuclear magnetic resonance structure of human immunodeficiency virus type 1 (HIV1) trans-activation response element (TAR) RNA in complex with acetopromazine. Black arrows mark acetopromazine (in green) and the nucleotides (in magenta) forming the UCU-bulge (see panel A).

To further test the influence of the *Eco* RPR_wt_ S domain with respect to phenothiazine inhibition, we determined the *K_i_* values for inhibition of two variants, *Eco* RPR_G235_ and *Eco* CP RPR, by toluidine blue O and chlorpromazine (the latter was not tested for inhibition of *Eco* CP RPR). *Eco* RPR_G235_ carries a mutation in the region near lead(II)-induced cleavage sites Ia, Ib, IIa and IIb (Figure 2; see also Figure 6) and it influences cleavage at sites Ia and IIb (Figure 5, cf. lane 9 vs. 4; [25]). The other variant *Eco* CP RPR lacks the S domain [30,31]. With pSu1 as substrate, toluidine blue O inhibited the activity of these two *Eco* RPR variants with similar *K_i_* values as observed for *Eco* RPR_wt_. For *Eco* RPR_G235_ and chlorpromazine, the *K_i_* was roughly twofold lower compared to that determined for *Eco* RPR_wt_ (Table 2). Moreover, the Pb^2+^-induced cleavage pattern of *Eco* RPR_G235_ in the presence of these two phenothiazine derivatives was similar compared to that observed for *Eco* RPR_wt_. Of note, while cleavage at site Ia for *Eco* RPR_G235_ was reduced in the absence of inhibitor, the addition of either toluidine blue O or chlorpromazine led to an even greater reduction in cleavage at that site (Figure 5, cf. lane 9 (without inhibitor) and lanes 7 and 10 (with inhibitor)). Together, these data suggest that both the S and C domains are targeted by phenothiazine derivatives (see also above).

## 3. Discussion

Various phenothiazine derivatives have been demonstrated to bind to RNAs (Figure 6). For example, the human immunodeficiency virus (HIV) trans-activation response element (TAR) RNA that interacts with the trans-activator of transcription (Tat) protein, forming a complex that is essential for activating the transcription of the HIV genome [35,36,37]. Moreover, nuclear magnetic resonance (NMR) data revealed binding of the phenothiazine acetopromazine [38] to small RNAs representing the *E. coli* ribosomal A site, the coxsackievirus (CV) B3 loop D RNA (CV loop D RNA), and the loop B of the poliovirus (PV) internal ribosome entry site (IRES) (PV loop B RNA). The *K_D_* values for HIV-TAR RNA, ribosomal A site, and CV loop D RNA were reported to be ≈300 μM whereas the *K_D_* for the PV loop B RNA, at 1.8 mM, was much higher [38]. Here, we provide data showing that different phenothiazines and pyronine G inhibited the activities of three structurally different bacterial RPRs, including *M. tuberculosis* RPR, while the three-ring compounds imipramine and amitriptyline did not. Interestingly, three antipsychotic phenothiazines known to have antibacterial activities [39] also inhibited the activity of all three bacterial RPRs; while their *K_i_* values were higher than those of the other phenothiazines tested, they were in the same range as the *K_D_* value for acetopromazine binding to, e.g., HIV-TAR RNA (Table 1; [38]). Collectively, our findings are, to the best of our knowledge, the first to show that long functional non-coding RNAs (≈400 nt) are targeted by different phenothiazine derivatives.

### 3.1. Comparison with Aminoglycoside Interaction with RNA

The *K_i_* values for the phenothiazines are similar to those determined for inhibition of *Eco* RPR_wt_ with and without the C5 protein by aminoglycosides such as neomycin B [12,15]. However, in contrast to aminoglycosides (and other RNA-based activities [40,41]) inhibition of bacterial RPR activity by phenothiazines does not appear to be pH-dependent. We also did not detect as pronounced an effect on inhibition in response to increasing Mg^2+^ and NH_4_^+^ concentrations as was reported for aminoglycoside derivatives [15,17]. At most, a fourfold increase in *K_i_* was observed (Table 3). Electrostatics is a major factor that accounts for aminoglycoside binding to RNA, and for *E. coli* and human rRNA A sites, the electrostatic contribution is about one-half of the total binding energy [42]. Contributions also come from non-ionic and pseudo-base-pair interactions, water-mediated contacts, shape complementarity, and conformational adaptation [15,17,40,41]. Moreover, NMR studies revealed that the interaction between TAR RNA and the aminoglycoside neomycin B results in distortion of the RNA structure, whereas binding of acetopromazine to TAT RNA induces only minor changes [43]. Hence, these differences suggest that phenothiazine binding to RNA is governed by features other than aminoglycoside binding [41]. However, as discussed below, structural information on phenothiazine RNA interaction is limited.

### 3.2. Phenothiazines Interact Preferentially with Internal RNA Bulges

Although, we do not know the mode of interaction of phenothiazines with bacterial RPRs, the NMR structure of the acetopromazine–TAR RNA complex and the phenothiazine’s interaction with other RNA molecules such as the *E. coli* A site RNA provide several insights (Figure 6; [36,38,43]). First, the *K_D_* values for acetopromazine binding to HIV-TAR RNA, *E. coli* A site RNA, and CV D loop RNA are reported to be approximately 300 μM. This is within the same range as the *K_i_* values for RPR inhibition by chlorpromazine, thioridazine, and trifluoperazine (Table 1), while the values for the methylene derivatives were significantly lower (Table 1). Second, phenothiazines have a nonplanar structure, and NMR data suggest that the tricyclic ring system is in closest contact with the RNA and forms most of the contacts, whereas weaker contacts are contributed by the aliphatic side chain. Third, acetopromazine has a preference for internal bulges and binding to smaller (two to three nucleotides in size) bulges is slightly stronger. Moreover, NMR data suggest only minor differences in the RNA structure with and without the inhibitor. Fourth, acetopromazine binds poorly to RNA loop structures and no detectable binding to a UUCG-tetraloop has been reported [38]. Fifth, there is no preference for internal bulges composed of purines or pyrimidines. Sixth, the identity of the 2-position substituent affects binding affinity. Finally, acetopromazine has a preference for internal bulges and hence it is likely that phenothiazines bind in the vicinity of bulges and internal loop regions in RPRs.

### 3.3. Phenothiazine Interaction with the S Domain and the Pre-tRNA D/T Loop

In the *Eco* RPR S domain, there are two internal bulges (Figure 2). Lead(II)-induced cleavage in the presence of toluidine blue O (and chlorpromazine) was affected at and near sites Ib, IIa, IIb and IIb′ in the S domain, which are proximal to these two internal bulges. The crystal structure of the type A RPR from *Thermus thermophilus* showed that sites Ia, Ib, IIa, IIb and IIb′ are in close proximity to each other ([44,45]; see also [34]). Moreover, the C to G change at position 235 in *Eco* RPR, which is located in the vicinity of these sites in the S domain (Figure 2), resulted in a twofold lower *K_i_* value for chlorpromazine while the *K_i_* of toluidine blue O was unaffected (Table 2). In addition, the lead(II)-induced cleavage patterns for *Eco* RPR_wt_ and *Eco* RPR_G235_ with and without phenothiazines were similar except for site Ia, in particular, which is known to be affected in response by having G at position 235 [25]. Taken together and based on the preference of acetopromazine for internal bulges, it is likely that phenothiazines bind at or in the vicinity of the bulges near site Ia, Ib, IIa, IIb, and IIb′ in the S domain. The S domains of type A and type B RPRs are similar [44] and type B *Hyo* RPR is cleaved by Pb^2+^ at a site in the S domain that corresponds to site Ia in *Eco* RPR ([26]; see also [46,47]). It is therefore reasonable to suggest that phenothiazines also bind the corresponding region(s) (i.e., near site Ia) in *Hyo* RPR. This putative phenothiazine binding site in the S domain would be close to the site that interacts the pre-tRNA T stem/loop (TSL) region, referred to as the TSL-binding site (TBS) [25]. Thus, binding of phenothiazine at (or near) this location might interfere with the interaction between TSL and TBS.

Our analysis further revealed that phenothiazines interact with yeast-tRNA^Phe^ and inhibit lead(II)-induced cleavage in the D loop between residues 16 and 17. Residues in the TSL region are involved in coordinating the Pb^2+^ ion that activates the 2′OH at position 16 that results in hydrolysis of the RNA [48,49]. The phenothiazines may also bind in the TSL region. However, neomycin B inhibition of lead(II)-induced cleavage of yeast tRNA^Phe^ is the result of binding distant to the cleavage site [18]. Thus, we cannot exclude that the phenothiazine binds elsewhere and indirectly interferes with the structural topology of the T/D loop such that binding of the catalytic Pb^2+^ is affected. Interestingly, pre-incubating phenothiazine and pre-tRNA prior to adding *Eco* RPR resulted in a four- to eightfold reduction in *K_i_* for toluidine blue O and thionine acid, respectively (Table 3). This indicates that inhibitor binding to the pSu3 substrate is more inhibitory than inhibitor binding to the RPR, which may be due to a structural change in the precursor tRNA that results in enhanced affinity for the inhibitor. However, inhibition of lead(II)-induced cleavage of yeast tRNA^Phe^ required higher phenothiazine concentrations compared to inhibition of RPR (Table 1). This might reflect differences in the pSu3 and yeast tRNA^Phe^ structures, which affect the binding affinities for the phenothiazines. Nevertheless, these observations raise the question that binding of some phenothiazine derivatives like chlorpromazine might interfere with the interaction between the TSL and TBS [25,50]. Additional studies are required to address these possibilities.

### 3.4. Phenothiazine Interaction with the C Domain and the Pre-tRNA D/T Loop

*Eco* CP RPR, which lacks the S domain, is still catalytic [30,31] and addition of toluidine blue O inhibited the activity (Table 2). This finding suggested that phenothiazines also bind to one or more other sites in *Eco* RPR in addition to the binding site in the S domain. Moreover, binding of Pb^2+^ ions in the P15–P17 region promotes hydrolysis at sites III, IVa, and V in full-length *Eco* RPR and the addition of chlorpromazine in particular influenced cleavage at these sites (Figure 2 and Figure 5; [51,52]). Lead(II)-induced cleavage at sites III and V was also detected using P15 RNA, a model representing the L15 internal loop (Figure 2; [32,33]); addition of phenothiazines inhibited lead(II)-induced cleavage of this RNA (Table 1). This suggests that phenothiazines bind the L15-loop of bacterial type A RPR, the region that interacts with the 3′RCC end of precursor tRNA [1,2] Thus, we conclude that phenothiazines interact with functionally important regions in both the S and C domains of bacterial RPRs. In this context it is of interest that both the L15 and TBS regions have been exploited as part of antisense approaches to inhibit the activity of bacterial RNase P [13,53,54,55].

### 3.5. Phenothiazine, a Scaffold with Potential

Phenothiazines are promiscuous and they bind and interfere with targets such as nitric oxide synthase, acetylcholinesterases, disulfide reductases, DNA, Ca^2+^-dependent enzymes, enzymes involved in lipid metabolism, ATP transporters, and various bacterial efflux pumps, and it has been suggested that trifluoperazine acts as a calmodulin antagonist [21,39,56]. Our present data adds RNA, particularly bacterial RPRs, to the list of phenothiazine targets. Comparing chlorpromazine inhibition of bacterial RPR (Table 1) and chlorpromazine inhibition of previously reported targets such as trypanothione reductase (*K_i_* = 10.8 μM [57]), Na^+^/K^+^-ATPase from rat brain (IC_50_ = 29 μM [58]) and mitochondrial malate dehydrogenase (inhibition observed at >140 μM [59]) reveals that higher concentrations are required to inhibit bacterial RPR. It should also be noted that since the discovery of methylene blue’s antimalarial activity, other phenothiazines have been synthesized and shown to be useful drugs in various settings. The best-known examples are the antipsychotic drugs chlorpromazine and thioridazine. These drugs also have antibacterial activity; in particular, thioridazine has apparently been instrumental in treating patients infected with extreme drug-resistant *M. tuberculosis* strains [60,61,62]. Given the structural differences between human and bacterial RNase P and the inhibition of bacterial RPRs, including *M. tuberculosis* RPR, by selected phenothiazine derivatives, development of new phenothiazine derivatives that are more specific in targeting bacterial RNase P warrants consideration. Additionally, it has been discussed that methylene blue might slow down the development of Alzheimer’s disease since it can inhibit the aggregation of tau protein ([21] and references therein). In this context, aminoglycosides have been reported to interact with the tau exon 10 splicing regulatory RNA element [63]. Therefore, this raises the possibility that phenothiazines also interfere with the expression of the tau protein and RNA splicing in general.

## 4. Materials and Methods

### 4.1. Preparation of RNA

The tRNA^Tyr^Su3 (pSu3) and tRNA^Ser^Su1 (pSu1) precursors were prepared, labeled internally with α-[^32^P]-UTP (pSu3) or 5′-labeled with γ-[^32^P]-ATP using T4 polynucleotide kinase (pSu1). The labeled precursor tRNAs were gel-purified as described in detail elsewhere [32,64,65,66]. The different bacterial RPRs, *E. coli* (*Eco* RPR_wt_, *Eco* RPR_G235_, and *Eco* CPR PR), *M. tuberculosis* (*Myc* RPR), and *Mycoplasma hyopneumoniae* (*Hyo* RPR) were generated as run-off transcripts using T7 DNA-dependent RNA polymerase as described previously [25,26,27,64,67].

### 4.2. Preparation of Compounds

The different phenothiazine derivatives, pyronine G, imipramine, and amitriptyline used in the present study were purchased from Sigma-Aldrich (Stockholm, Sweden) and VWR International (Stockholm, Sweden). All compounds were dissolved in double-distilled water prior to use.

### 4.3. RNase P Assay Conditions

RNase P RNA (RPR) activity with and without inhibitors was monitored as described elsewhere [15] in buffer I (50 mM Tris·HCl, pH 7.9/5% (*w/v*) polyethylene glycol 6000/100 mM NH_4_Cl/100 mM MgCl_2_) and buffer II (50 mM Tris·HCl, pH 7.9/5% (*w/v*) polyethylene glycol 6000/1 mM NH_4_Cl/10 mM spermidine/10 mM MgCl_2_). The reaction products were separated on denaturing polyacrylamide gels (8% (*w/v*) and 25% (*w/v*) for pSu3 and pSu1, respectively) and visualized with a PhosphorImager. The final concentrations of substrate and RPR (*Eco* RPR_wt_, *Eco* RPR_G235_, *Myc* RPR and *Hyo* RPR) were ≤20 nM and 82 nM, respectively. In the case of *Eco* CP RPR 5.4 μM was used.

### 4.4. Lead(II)-Ion Induced Cleavage of Yeast tRNA^Phe^, Eco RPR and P15 RNA

*Eco* RPR and yeast tRNA^phe^ were 3′ end-labeled with [^32^P]-pCp following standard procedures [68]. The 31 nucleotides long P15 RNA was 5′ end-labeled with γ-[^32^P]-ATP as described elsewhere [32]. Lead (II)-induced cleavage was done in 50 mM Tris–HCl (pH 7.5), 1 mM NH_4_Cl; 10 mM MgCl_2_ as previously described [15]. The final concentration of Pb(OAc)_2_ was 0.5 mM while the concentrations of toluidine blue O and chlorpromazine were as indicated. For additional details see Kirsebom and Ciesiolka [68].

### 4.5. Determination of the Inhibition Constant *K_i_*

The *K_i_* value was defined as the inhibitor concentration that results in 50% inhibition of cleavage. Conditions were adjusted so that measurements were carried out when product formation was in the linear part of the curve of kinetics of the cleavage reaction. Each value in μM was calculated from plots as the one in Figure 4 and the values are averages of three independent experiments ± the maximum deviation of the average value.

## Figures and Tables

**Table 1:** *K_i_* values (in μM) for inhibition of *Eco* RPR, *Myc* RPR and *Hyo* RPR mediated cleavage of pre-tRNA^Tyr^Su3 (pSu3) and lead(II)-induced cleavage of P15 RNA and yeast tRNA^Phe^.

	RPR-Mediated Cleavage			Lead(II)-Induced Cleavage	
	*Eco*	*Myc*	*Hyo*	P15 RNA	Yeast tRNA^Phe^
Methylene blue	14 ± 2.6	28 ± 13	25 ± 1.2	62 ± 6.3	98 ± 20
Toluidine blue O	8.6 ± 1.7	12 ± 3.8	10 ± 4.9	14 ± 8	70 ± 20
Thionine acid	17 ± 0.33	8.3 ± 4.3	9.3 ± 3.3	41 ± 1.3	46 ± 51
Azure A	29 ± 9	21 ± 2.3	19 ± 0.67	28 ± 8.3	52 ± 8.3
Azure B	17 ± 9	12 ± 2	12 ± 2.7	37 ± 10	58 ± 28
Pyronin G	15 ± 1.7	44 ± 7.0	35 ± 8.6	20 ± 3	166 ± 42
Thioridazine	177 ± 23	67 ± 6.2	125 ± 31	80 ± 1.7	ND
Trifluoperazine	131 ± 22	106 ± 3.3	151 ± 26	132 ± 24	ND
Chlorpromazine	437 ± 33	161 ± 16	170 ± 9.2	133 ± 19	ND

*K_i_* corresponds to the concentration that led to 50% inhibition of cleavage activities. Each value was calculated from plots like the one in Figure 4 and represents the average of three independent experiments ± the maximum deviation from the average value. The concentration of pSu3 was ≤0.02 μM while the concentrations of the RPR variants, P15 RNA, and yeast tRNA^Phe^ were 0.082 μM, 5 μM, and 2.4 μM, respectively (for details see Materials and Methods). *Eco*: *Escherichia coli*; *Myc*: *Mycobacterium tuberculosis*; *Hyo*: *Mycoplasma hyopneumoniae*; RPR: RNase P RNA; ND: not determined.

**Table 2:** *K_i_* values (in μM) for inhibition of cleavage of pre-tRNA^Ser^Su1 (pSu1) by different *Eco* RPR variants.

*Eco* RPR Variant	Toluidine Blue O (μM)	Chlorpromazine (μM)
*Eco* RPR_wt_	20 ± 4	450 ± 40
*Eco* RPR_G235_	17 ± 3	200 ± 30
*Eco* CP RPR	30 ± 5	ND

*K_i_* corresponds to the concentration that led to 50% inhibition of cleavage activities by the indicated *Eco* RPR variants. Each value was calculated from plots like the one in Figure 4 and represents the average of three independent experiments ± the maximum deviation from the average value. The concentration of pSu1 was ≤0.02 μM while the concentrations of *Eco* RPR_wt_ and *Eco* RPR_G235_ were 0.082 μM and *Eco* CP RPR was 5.4 μM (for details see Materials and Methods). ND, not determined.

**Table 3:** *K_i_* values (in μM) for inhibition of *Eco* RPR_wt_ mediated cleavage under different conditions.

Inhibitor	Conditions	Order of Addition	*K_i_* Values (μM)
Toluidine blue O	Buffer I, pH 7.2	RPR + I then S	16 ± 2
Toluidine blue O	Buffer II, pH 7.2	RPR + I then S	8.6 ± 0.9
Toluidine blue O	Buffer II, pH 9.0	RPR + I then S	11 ± 2.6
Toluidine blue O	Buffer II, pH 6.0	RPR + I then S	9.3 ± 0.67
Toluidine blue O	Buffer II, pH 7.2	RPR, I and S	10 ± 1.8
Toluidine blue O	Buffer II, pH 7.2	S + I then RPR	2.2 ± 0.4
Thionine acid	Buffer I, pH 7.2	RPR + I then S	63 ± 6.8
Thionine acid	Buffer II, pH 7.2	RPR + I then S	17 ± 0.3
Thionine acid	Buffer II, pH 7.2	RPR, I and S	7.5 ± 3.5
Thionine acid	Buffer II, pH 7.2	S + I then RPR	2.2 ± 0.4

RPR + I then S: RPR + I were pre-incubated for 10 min at 37 °C followed by the addition of S; S + I then RPR: S + I were pre-incubated for 10 min at 37 °C followed by the addition of pre-incubated *Eco* RPR_wt_; RPR, I and S: RPR, I and S were pre-incubated at 37 °C separately for 10 min and then mixed. *K_i_* corresponds to the concentration that led to 50% inhibition of cleavage activities as indicated. Each value was calculated from plots like the one in Figure 4 and represents the average of three independent experiments ± the maximum deviation from the average value. The concentration of pSu3 was ≤0.02 μM while the concentration of *Eco* RPR was 0.082 μM.

## References

[1] Kirsebom L.A., Trobro S. (2009). RNase P RNA-mediated cleavage. IUBMB Life.

[2] Liu F., Altman S. (2010). Ribonuclease P (Protein Reviews).

[3] Lai L.B., Vioque A., Kirsebom L.A., Gopalan V. (2010). Unexpected diversity of RNase P, an ancient tRNA processing enzyme: Challenges and prospects. FEBS Lett..

[4] Guerrier-Takada C., Gardiner K., Marsh T., Pace N., Altman S. (1983). The RNA moiety of ribonuclease P is the catalytic subunit of the enzyme. Cell.

[5] Bartkiewicz M., Gold H., Altman S. (1989). Identification and characterization of an RNA molecule that copurifies with RNase P activity from HeLa cells. Genes Dev..

[6] Jarrous N., Gopalan V. (2010). Archaeal/eukaryal RNase P: Subunits, functions and RNA diversification. Nucl. Acids Res..

[7] Kirsebom L.A., Eggelston D.S., Prescott C.D., Pearson N.D. (1998). RNase P and its substrate. The Many Faces of RNA.

[8] Kirsebom L.A., Barciszewski J., Clark B.F.C. (1999). The structure and function of the ribozyme RNase P RNA is dictated by magnesium(II) ions. RNA Biochemistry and Biotechnology.

[9] Kirsebom L.A., Svärd S.G., Krupp G., Gaur R.K. (2000). RNase P processing of tRNA precursors. Ribozyme Biochemistry and Biotechnology.

[10] Kirsebom L.A., Virtanen A., Schroeder R., Wallis M.G. (2001). Inhibition of RNase P processing. RNA-Binding Antibiotics.

[11] Eder P.S., Hatfield C., Vioque A., Gopalan V. (2003). Bacterial RNase P as a potential target for novel anti-infectives. Curr. Opin. Investig. Drugs.

[12] Kirsebom L.A., Virtanen A., Mikkelsen N.E. (2006). Aminoglycoside interactions with RNAs and nucleases. Handb. Exp. Pharmacol..

[13] Willkomm D.K., Pfeffer P., Reuter K., Klebe G., Hartmann R.K., Liu F., Altman S. (2010). RNase P as a drug target. Ribonuclease P.

[14] Vioque A. (1989). Protein synthesis inhibitors and catalytic RNA. Effect of puromycin on tRNA precursor processing by the RNA component of *Escherichia coli* RNase P. FEBS Lett..

[15] Mikkelsen N.-E., Brännvall M., Virtanen A., Kirsebom L.A. (1999). Inhibition of RNase P RNA cleavage by aminoglycosides. Proc. Natl. Acad. Sci. USA.

[16] Eubank T.D., Biswas R., Jovanovic M., Litovchick A., Lapidot A., Gopalan V. (2002). Inhibition of bacterial RNase P by aminoglycosides-arginine conjugates. FEBS Lett..

[17] Kawamoto S.A., Sudhahar C.G., Hatfield C.L., Sun J., Behrman E.J., Gopalan V. (2008). Studies on the mechanism of inhibition of bacterial ribonuclease P by aminoglycoside derivatives. Nucleic Acids Res..

[18] Mikkelsen N.-E., Johansson K., Virtanen A., Kirsebom L.A. (2001). Aminoglycoside binding displaces a divalent metal ion in a tRNA-neomycin B complex. Nat. Struct. Biol..

[19] Papadimou E., Georgiou S., Tsambaos D., Drainas D. (1998). Inhibition of ribonuclease P activity by retinoids. J. Biol. Chem..

[20] Tekos A., Tsagla A., Stathopoulos C., Drainas D. (2000). Inhibition of eukaryotic ribonuclease P activity by aminoglycosides: Kinetic studies. FEBS Lett..

[21] Schirmer R.H., Adler H., Pickhardt M., Mandelkow E. (2011). “Lest we forget you—Methylene blue…”. Neurobiol. Aging.

[22] Spicer S.S. (1961). Differentiation of nucleic acids by staining at controlled pH and by a Schiff-methylene blue sequence. Strain Technol..

[23] Wilkinson M., Doskow J., Lindsey S. (1991). RNA blots: Staining procedures and optimization of conditions. Nucleic Acids Res..

[24] Haas E.S., Brown J.W. (1998). Evolutionary variation in bacterial RNase P RNAs. Nucleic Acids Res..

[25] Brännvall M., Kikovska E., Wu S., Kirsebom L.A. (2007). Evidence for induced fit in bacterial RNase P RNA-mediated cleavage. J. Mol. Biol..

[26] Svärd S.G., Mattsson J.G., Johansson K.-E., Kirsebom L.A. (1994). Cloning and characterization of the RNase P RNA genes from two porcine mycoplasmas. Mol. Microbiol..

[27] Svärd S.G., Kagardt U., Kirsebom L.A. (1996). Phylogenetic comparative mutational analysis of the base-pairing between RNase P RNA and its substrate. RNA.

[28] Dooley K.E., Phillips P.P., Nahid P., Hoelscher M. (2016). Challenges in the clinical assessment of novel tuberculosis drugs. Adv. Drug Deliv. Rev..

[29] Massire C., Jaeger L., Westhof E. (1998). Derivation of the three-dimensional architecture of bacterial ribonuclease P RNAs from comparative sequence analysis. J. Mol. Biol..

[30] Kikovska E., Wu S., Mao G., Kirsebom L.A. (2012). Cleavage mediated by the P15 domain of bacterial RNase P RNA. Nucleic Acids Res..

[31] Wu S., Kikovska E., Lindell M., Kirsebom L.A. (2012). Cleavage mediated by the catalytic domain of bacterial RNase P RNA. J. Mol. Biol..

[32] Kufel J., Kirsebom L.A. (1998). The P15-loop of *Escherichia coli* RNase P RNA is an autonomous divalent metal ion binding domain. RNA.

[33] Glemarec C., Kufel J., Földesi A., Maltseva T., Sandström A., Kirsebom L.A., Chattopadhyaya J. (1996). The NMR structure of 31mer RNA domain of *Escherichia coli* RNase P RNA using its non-uniformity deuterium labelled counterpart [the ‘NMR-window’ concept]. Nucleic Acids Res..

[34] Reiter N.J., Osterman A., Torres-Larios A., Swinger K.K., Pan T., Mondragón A. (2010). Structure of a bacterial ribonuclease P holoenzyme in complex with tRNA. Nature.

[35] Lind K.E., Du Z., Fujinaga K., Peterlin B.M., James T.L. (2002). Structure-based computational database screening in vitro assay, and NMR assessment of compounds that target TAR RNA. Chem. Biol..

[36] Du A., Lind K.E., James T.L. (2002). Structure of TAR RNA complexed with a Tat-TAR interaction nanomolar inhibitor that was identified by computational screening. Chem. Biol..

[37] Mayer M., Lang T., Gerber S., Madrid P.B., Gómez Pinto I., Guy R.K., James T.L. (2006). Synthesis and testing of a focused phenothiazine library for binding to HIV-1 TAR RNA. Chem. Biol..

[38] Mayer M., James T.L. (2004). NMR-based characterization of phenothiazines as a RNA binding scaffold. J. Am. Chem. Soc..

[39] Kristiansen J.E., Dastidar S.G., Palchoudhuri S., Sinha Roy D., Das S., Hendricks O., Christensen J.B. (2015). Phenothiazines as a solution for multidrug resistant tuberculosis: From the origin to present. Int. Microbiol..

[40] Hermann T., Westhof E. (1998). Aminoglycoside binding to the hammerhead ribozyme: A general model for the interaction of cationic antibiotics with RNA. J. Mol. Biol..

[41] Thomas J.R., Hergenrother P.J. (2008). Targeting RNA with small molecules. Chem. Rev..

[42] Kaul M., Barbieri C.M., Pilch D.S. (2005). Defining the basis for the specificity of aminoglycoside-rRNA recognition: A comparative study of drug binding to the A sites of *Escherichia coli* and Human rRNA. J. Mol. Biol..

[43] Pitt S.W., Zhang Q., Patel D.J., Al-Hashimi H.M. (2005). Evidence that electrostatic interactions dictate the ligand-induced arrest of RNA global flexibility. Angew. Chem. Int. Ed..

[44] Krasilnikov A.S., Xiao Y., Pan T., Mondragón A. (2004). Basis for structural diversity in homologous RNAs. Science.

[45] Lindell M., Brännvall M., Wagner E.G.H., Kirsebom L.A. (2005). Lead(II) cleavage analysis of RNase P RNA in vivo. RNA.

[46] Tallsjö A., Kufel J., Kirsebom L.A. (1993). A novel tertiary interaction in M1 RNA, the catalytic subunit of *Escherichia coli* RNase P. Nucleic Acids Res..

[47] Zito K., Hüttenhofer A., Pace N.R. (1993). Lead-catalyzed cleavage of ribonuclease P RNA as a probe for integrity of tertiary interaction. Nucleic Acids Res..

[48] Brown R.S., Dewan J.C., Klug A. (1985). Crystallographic and biochemical investigation of the lead(II)-catalyzed hydrolysis of yeast phenylalanine tRNA. Biochemistry.

[49] Behlen L.S., Sampson J.R., DiRenzo A.B., Uhlenbeck O.C. (1990). Lead-catalyzed cleavage of yeast tRNA^Phe^ mutants. Biochemistry.

[50] Loria A., Pan T. (1997). Recognition of the T stem-loop of a pre-tRNA substrate by the ribozyme from *Bacillus subtilis* ribonuclease P. Biochemistry.

[51] Ciesiolka J., Hardt W.D., Schlegl J., Erdmann V.A., Hartmann R.K. (1994). Lead-ion-induced cleavage of RNase P RNA. Eur. J. Biochem..

[52] Brännvall M., Mikkelsen N.-E., Kirsebom L.A. (2001). Monitoring the structure of *Escherichia coli* RNase P RNA in the presence of various divalent metal ions. Nucleic Acids Res..

[53] Childs J.L., Poole A.W., Turner D.H. (2003). Inhibition of *Escherichia coli* RNase P by oligonucleotide directed misfolding of RNA. RNA.

[54] Gruegelsiepe H., Willkommen D.K., Goudinakis O., Hartmann R.K. (2003). Antisense inhibition of *Escherichia coli* RNase P RNA: Mechanistic aspects. ChemBioChem.

[55] Gruegelsiepe H., Brandt O., Hartmann R.K. (2006). Antisense inhibition of RNase P: Mechanistic aspects and application to live bacteria. J. Biol. Chem..

[56] Ratnakar P., Murthy P.S. (1993). Trifluoperazine inhibits the incorporation of labelled precursors into lipids, proteins and DNA of *Mycobacterium tuberculosis* H37RV. FEMS Microbiol. Lett..

[57] Khan M.O., Austin S.E., Chan C., Yin H., Marks D., Vaghjiani S.N., Kendrick H., Yardley V., Croft S.L., Douglas K.T. (2000). Use of an additional hydrophobic binding site, the Z site, in the rational drug design of a new class of stronger trypanothione reductase inhibitor, quarternary alkylammonium phenothiazines. J. Med. Chem..

[58] Carfagna M.A., Muhoberac B.B. (1993). Interaction of tricyclic drug analogs with synaptic plasma membranes: Structure-mechanism relationships in inhibition of neuronal Na+/K(+)-ATPase activity. Mol. Pharmacol..

[59] Chowdhury A.K., Rogers H., Skinner A., Spector R.G., Watts D.C. (1969). The influence of psychotropic drugs on aldolase, mitochondrial malic dehydrogenase and Mg^++^Na^+^K^+^ adenosine triphosphate. Br. J. Pharmacol..

[60] Amaral L., Martins A., Molnar J., Kristiansen J.E., Martins M., Viveiros M., Rodrigues L., Spenglier G., Couto I., Ramos J. (2010). Phenothiazines, bacterial efflux pumps and targeting the macrophage for enhanced killing of intracellular XDRTB. In Vivo.

[61] Van Soolingen D., Hernandez-Pando R., Orozco H., Aguilar D., Magis-Escurra C., Amaral L., van Ingen J., Boeree M.J. (2010). The antipsychotic thioridazine shows promising therapeutic activity in a mouse model of multidrug-resistant tuberculosis. PLoS ONE.

[62] Abbate E., Vescovo M., Natiello M., Cufré M., Garcia A., Gonzalez Montaner P., Ambroggi M., Ritacco V., van Soolingen D. (2012). Successful alternative treatment of extensively drug-resistant tuberculosis in Argentina with a combination of linezolid, moxifloxacin and thioridazine. J. Antimicrob. Chemother..

[63] Varani L., Spillantini M.G., Goedert M., Varani G. (2000). Structural basis for recognition of the RNA major groove in the tau exon 10 splicing regulatory element by aminoglycoside antibiotics. Nucleic Acids Res..

[64] Milligan J.F., Groebe D.R., Whiterell G.W., Uhlenbeck O.C. (1987). Oligoribonucleotide synthesis using T7 RNA polymerase and synthetic DNA templates. Nucleic Acids Res..

[65] Kirsebom L.A., Svärd S.G. (1993). Identification of a region within M1 RNA of *Escherichia coli* RNase P important for the location of the site of cleavage on a wild-type tRNA precursor. J. Mol. Biol..

[66] Brännvall M., Kirsebom L.A. (1999). Manganese ions induce miscleavage in the *Escherichia coli* RNase P RNA-catalyzed reaction. J. Mol. Biol..

[67] Vioque A., Arnez J., Altman S. (1988). Protein-RNA interactions in the RNase P holoenzyme from *Escherichia coli*. J. Mol. Biol..

[68] Kirsebom L.A., Ciesiolka J., Hartmann R.K., Bindereif A., Schön A., Westhof E. (2014). Pb^2+^-Induced Cleavage of RNA. Handbook of RNA Biochemistry.

